# Identification of a novel lactose oxidase in *Myrmecridium flexuosum* NUK‐21

**DOI:** 10.1002/2211-5463.12582

**Published:** 2019-01-16

**Authors:** Shuen‐Fuh Lin, Cheng‐Ke Li, Yi‐Pei Chung

**Affiliations:** ^1^ Department of Life Sciences National University of Kaohsiung Taiwan

**Keywords:** lactobionic acid, lactose oxidase, *Myrmecridium flexuosum*, Zn cofactor

## Abstract

Lactobionic acid (*O*‐β‐galactosyl‐(1‐4)‐gluconic acid) (LBA) is a high‐value lactose derivative, produced via oxidation of the reducing terminal of lactose. LBA can be produced by fermentation using certain microorganisms, although subsequent purification is challenging. Therefore, we have attempted to identify an enzyme for possible use in LBA production. Here, we purified a novel lactose oxidase (LOD) to homogeneity from a wheat bran culture of a soil‐isolated fungal strain, *Myrmecridium flexuosum* NUK‐21. Maximal activity was observed on the wheat bran solid culture after 3 days of NUK‐21 growth, following release from cells at 0.66 unit·mL ^−1^ culture filtrate. This new sugar oxidase was composed of a single polypeptide chain with a molecular mass of 47.2 kDa and was found to contain 2.0 zinc ions per mole of enzyme but no flavin adenine dinucleotide or heme. This enzyme was stable in the pH range 5.5–9.0, with an optimal reaction pH of 7.5. Its optimal reaction temperature was 40 °C, and it was stable up to 50 °C for 1 h at pH 7.5. LOD oxidized disaccharides with reducing‐end glucosyl residues linked by an α or β‐1,4 glucosidic bond. The relative activity of LOD toward lactose, cellobiose and maltose was 100 : 83 : 4, respectively. To the best of our knowledge, this is the first report on the discovery of an LOD based on coenzyme moiety and enzyme substrate specificity.

**Abbreviations**
4AA: 4‐aminoantipyrine
CBG: cellobiose quinone dehydrogenase
COOX: cellooligosaccharide oxidase
COX: carbohydrate:acceptor oxidoreductase
DCPIP: 2,6‐dichlorophenol‐indophenol
FPLC: fast performance liquid chromatography
GOOX: glucooligosaccharide oxidase
ICP‐MS: inductively coupled plasma mass spectrometry
LBA: lactobionic acid (*O*‐β‐galactosyl‐(1‐4)‐gluconic acid)
LOD: lactose oxidase
PAGE: polyacrylamide gel electrophoresis
PCOX: carbohydrate:acceptor oxidoreductase from *Paraconniothyrium* sp.
TLC: thin‐layer chromatography

Enzymes that catalyze the oxidation of carbohydrates directly to corresponding aldonic acids appear to be widely distributed in nature; they have been obtained from bacteria, fungi, algae and animal tissues. Lactobionic acid (*O*‐β‐galactosyl‐(1‐4)‐gluconic acid) (LBA) is produced via oxidation of the reducing terminal of lactose. Lactobionic acid is a high‐value lactose derivative. It has been a popular research topic because of its application in the cosmetics, pharmaceutical, food and chemical industries 1. The market for LBA is between 15 000 and 17 000 tons per year, with an expected annual growth rate of 5%.

Several processes, including chemical, electrochemical, biocatalytic and heterogeneous catalytic oxidation, have been investigated for the production of LBA. In the early years, almost no suitable strain or enzyme could be used to produce lactobionic acid and, thus, most lactobionic acids were produced by chemical transformation; for example, oxidation of lactose by bromine in an aqueous buffered solution to produce lactobionic acid 2. Subsequently, many chemical processes to convert lactose to lactobionic acid, such as the electrochemical method, have been reported. However, this method also generates toxic by‐products and causes environmental pollution. The enzymatic process is advantageous because it avoids the poisonous chemicals produced by microbial fermentation.

LBA is produced by microbes, including *Pseudomonas taetrolens* 3, 4, 5, *Zymomonas mobilis* 6, 7, *Burkholderia cepacia* 8, 9 and *Acetobacter orientalis* 10. However, some high‐yielding strains are mutants, which may give rise to safety concerns. In addition, although fermentation by the aforementioned microorganisms can produce a broth containing lactobionic acid in a fermentor, the subsequent purification steps are difficult to conduct. Therefore, in the present study, an enzyme production method was proposed. We conducted the direct enzymatic oxidation of lactose to expand the application of LBA. This method is advantageous because the relatively pure lactose can be directly converted to lactobionic acid and, thus, the purification steps after the reactions can be substantially simplified or eliminated. Compared with the microbial fermentation method, the enzyme catalytic method yields LBA of high purity at cheaper costs.

Various sugar oxidoreductases have been identified from a variety of sources, including bacteria 11, fungi 12, 13 and algae 14, 15. Most of the identified sugar oxidoreductases are specific to monosaccharides. Only a few oxidoreductases possess high specificity toward oligosaccharides. These include galactose oxidase obtained from *Polyporus circinatus* 16, which exhibits high reactivity to oligosaccharides containing galactose end groups; cellobiose dehydrogenase (EC 1.1.99.18) and cellobiose quinone oxidoreductase obtained from *Phanerochaete chrysosporium* 17 and *Spotoyrichum pulverolentum* 18, which are specific to cellooligosaccharides; carbohydrate:acceptor oxidoreductase (COX) obtained from *Microdochium nivale* and *Sarocladium oryzae* that reacts with oligo and polymeric long‐chain carbohydrates 19, 20; and COX obtained from *Paraconiothyrium* sp., which are efficiently oxidized β‐(1‐4) linked sugars, such as lactose, xylobiose and cellooligosaccharides 21.

None of the sugar oxidases reported have exhibited the highest reactivity toward lactose, which is composed of β‐d‐glucopyranosyl units joined by a 1–4 bond. The relative activities of COX obtained from *M. nivale* 20 and COX obtained from *Paraconiothyrium* sp. 21 toward lactose and cellobiose were 100 : 192 and 100 : 121, respectively, whereas the enzymes described above contain only FAD as the prosthetic group.

Recently, we applied a rapid and convenient screening method for the *in situ* assay of sugar oxidase‐producing soil fungi in accordance with the description by Danneel *et al*. 22. Strain NUK‐21, identified as *M. flexuosum*, was isolated. Oxidase purified from aqueous extract of a wheat bran solid‐state culture of this strain contained Zn as the prosthetic group and exhibited the highest reactivity toward lactose. This new oxidase was named lactose oxidase (LOD) based on its substrate specificity and prosthetic group. LOD was sufficiently stable for use in lactose oxidation. Here, we report the purification and characterization of this novel LOD and discuss some of its properties.

## Materials and methods

### Chemicals

Fractogel DEAE‐650M, HW‐50 and HW‐55 gel filtration resins were purchased from Merck (Darmstadt, Germany). Toyopearl Phenyl‐650M was purchased from Toyo Soda Manufacturing (Tokyo, Japan). Ultragel‐HA was acquired from IBF Biotechnics (Pairs, France). Protein assay dye and SDS/PAGE molecular weight standards were obtained from Bio‐Rad (Hercules, CA, USA). Sephacryl S‐200 HR16/60 fast performance liquid chromatography (FPLC) column was obtained from GE Healthcare Bio‐Science (Uppsala, Sweden). FPLC molecular weight standards, 4‐aminoantipyrine (4AA), peroxidase and all the sugars were from Sigma Chemical Co. (St. Louis, MO, USA). All other chemicals were of analytic reagent grade.

### Microorganisms and culture conditions

The fungal strain NUK‐21 was isolated from the soil, identified as *M. flexuosum* and then deposited at the Bioresource Collection and Research Center (BCRC, Hsinchu, Taiwan) of Taiwan under accession number BCRC 930190. The organism was maintained on YM agar slants (1% glucose, 0.5% peptone, 0.3% malt extract and 0.3% yeast extract) at 30 °C. For enzyme production, the organism was grown in 1‐L flasks, each containing 100 g of wheat bran supplemented with 200 mL of water. After 5–7 days of cultivation at 30 °C, the entire culture was used for enzyme preparation.

### Enzyme assay

The LOD activity was estimated via the peroxidase‐4AA method. An aliquot of enzyme was incubated at 30 °C in 1 mL of 50 mm Tris–HCl buffer (pH 7.8, buffer A) containing 7 mm lactose, 2 U of peroxidase, 0.1 mm 4AA and 1 mm phenol. The increase in optical density was measured at 500 nm for 1 min. The enzyme activity was estimated by monitoring the consumption of oxygen with an oxygraph (Oxy‐5; Gilson Medical Electronic, Villiers le Bel, France). The reaction was initiated by the addition of appropriate amounts of the enzyme to the reaction mixture containing 10 mm lactose in buffer A, and the initial velocity of oxygen consumption was measured. One unit of enzyme activity was defined as the amount of enzyme that produced 1 μmol·min^−1^ H_2_O_2_ or consumed 1 μmol·min^−1^ O_2_ at 30 °C.

The dehydrogenase activity of LOD was also estimated by monitoring the reduction of 2,6‐dichlorophenol‐indophenol (DCPIP) at 600 nm (ε = 2.7 mm ^−1^·cm^−1^) in the presence of 5 mm substrate in 50 mm phosphate buffer (pH 6.0). One unit of enzyme activity was defined as the amount of enzyme that reduced 1 μmol·min^−1^ DCPIP at 30 °C.

### Enzyme purification

A typical purification scheme of the LOD from wheat bran culture extract is described below. All operations were conducted at 4 °C.

#### Preparation of crude extract

NUK‐21 (100 g) wheat bran culture was soaked in 1 L of 50 mm buffer A with 0.2% SDS for 30 min and squeezed through a fine mesh cloth. The aqueous extract was then centrifuged at 9000 ***g*** for 30 min to remove particles.

#### Ammonium sulfate fractionation

The clarified aqueous extract (800 mL) was brought to 55% saturation with ammonium sulfate; the precipitate was removed via centrifugation. The resulting filtrate was collected via centrifugation.

#### Toyopearl phenyl‐650M column chromatography

The 55% saturated ammonium sulfate filtrate was applied to a Toyopearl phenyl‐650M column (2.5 × 30 cm) (Toyo Soda Manufacturing), which was pre‐equilibrated with buffer A containing 55% saturated ammonium sulfate ammonium sulfate. LOD was eluted with a 600 mL linear gradient of 55–0% ammonium sulfate in buffer A.

#### Fractogel HW‐50 column chromatography

The enzyme solution was applied to a Fractogel HW‐50 column (2.5 × 115 cm) (Merck), which was pre‐equilibrated with 50 mm buffer A containing 0.2% SDS. To concentrate the pooled active fractions (350 mL), ammonium sulfate was added to the enzyme solution to a final concentration of 2 m and then recharged to a small Toyopearl phenyl‐650M column (1 cm × 6 cm), which was pre‐equilibrated with buffer A containing 2 m ammonium sulfate. In this process, the enzyme was eluted directly with buffer A. The volume of pooled active fractions was 15 mL.

#### Ultragel‐HA hydroxylapatite column chromatography

The pooled active fractions were further purified by applying them to an Ultragel‐HA hydroxylapatite column (3 cm × 15 cm) (IBF Biotechnics), which was pre‐equilibrated with 10 mm phosphate buffer (pH 7.0). The enzyme was eluted with a 1000 mL linear gradient of 10–400 mm phosphate buffer. The active fractions were pooled and stored at −20 °C.

### Other analytical methods

The molecular mass of the native enzyme was estimated via gel filtration under the conditions: system, FPLC system; pump, P‐500 (GE Healthcare, Little Chalfont, UK); controller, LCC‐500 (Pharmacia Biotech, Uppsala, Sweden); detection, absorbance at 280 nm; column, Sephacryl S‐200 HR16/60 FPLC column (GE Healthcare); solvent, 100 mm NaCl in 10 mm acetate buffer (pH 5.5). The molecular mass of the denatured enzyme was determined via SDS/PAGE on 10% acrylamide slabs using a modified Laemmli buffer system 23. For activity staining, proteins were separated on a non‐denatured 7% polyacrylamide gel, followed by overlaying the gel onto filter paper (Toyo Roshi Kaisha, Tokyo, Japan) soaked in an activity assay solution (10 mm lactose, 2 U of peroxidase, 0.1 mm 4AA and 1 mm phenol in buffer A) for 15 min at 30 °C. Protein with enzyme activity was detected as a red band on the filter paper. Periodic acid–Schiff staining for glycoprotein was performed as described by Zaccharius *et al*. 24.

Protein concentrations were determined via the Bradford method with bovine serum albumin as the standard 25. Protein assay reagents were purchased from Bio‐Rad.

The metal content (20 elements) in the enzyme was analyzed by plasma emission spectroscopy with an ELAN 6000 DRC II ICP‐MS instrument (PerkinElmer, Concord, ON, Canada). The sample introduction system was combined with inductively coupled plasma mass spectrometry (ICP‐MS) for zinc speciation analysis.

Nuclear magnetic resonance spectra were recorded on a AV 400 instrument (Bruker, Billerica, MA, USA) (400 MHz for ^1^H and 100 MHz for ^13^C) with D_2_O (deuterium oxide) as the solvent.

### Thin‐layer chromatography (TLC) analysis of the reaction product

A reaction mixture (1 mL) containing 2.5% lactose (w/v), 2 units of LOD, 10 units of catalase and 1 N NaOH, was incubated at 30 °C with a reciprocal shaker (120 rpm). Subsequently, the reaction product was detected using TLC with silica gel 60 plates (Merck). Aliquots (approximately 0.2 μL) of sample were applied to the TLC plate and developed in isopropanol/water (4 : 1, v/v). The plate was sprayed with 50% sulfuric acid dissolved in ethanol/water (1 : 1, v/v) and then heated at 150 °C until charring occurred.

## Results

### LOD purification

**Figure 1:**
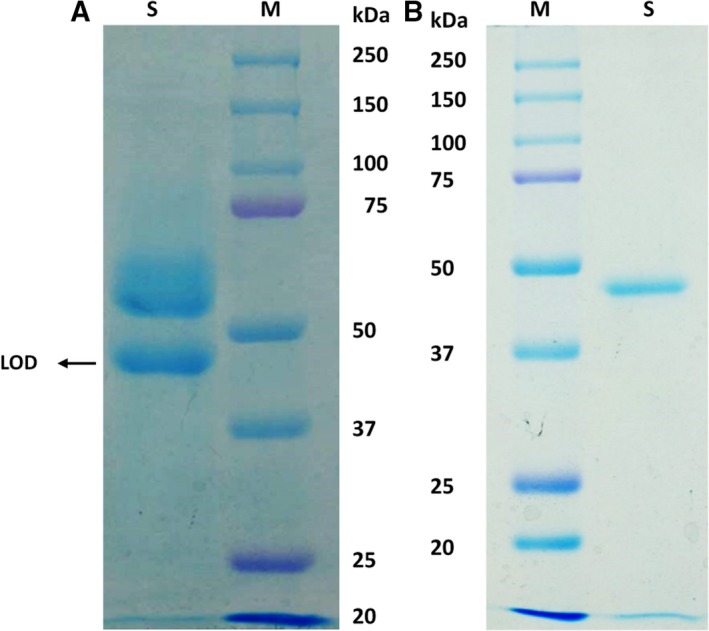
SDS/PAGE analysis of LOD. The gel concentration was 10%. (A) Lane M, molecular mass markers; lane S, 20 μg of the major aggregations of proteins after Fractogel HW‐50 column chromatography with 0.2% SDS. (B) Lane S, 6 μg of purified enzyme.

The *M. flexuosum* NUK‐21 strain produced LOD activity in both the wheat bran solid‐state culture and submerged culture. The productivity of the enzyme was similar. Maximal activity was observed after 3 days of growth in the wheat bran solid‐state culture and after 4 days in the submerged culture, with activities of 0.66 and 0.8 units·mL^−1^ culture filtrate, respectively. Thus, the enzyme was purified from the culture supernatant after a 3‐day wheat bran culture, as summarized in Table 1. A 7.6‐fold purification was obtained, with a recovery of 5.4%. The specific activity of the purified enzyme was 5.3 U·mg^−1^ protein. The enzyme activity decreased when the enzyme was subjected to Fractogel HW‐50 column chromatography with 0.2% SDS. This result might be attributed to the removal of some unknown proteins that can aggregate to LOD from the enzyme preparation in this step (Fig. 1A). This might be because of the removal of some unknown proteins that can aggregate to LOD from the enzyme preparation in this step (Fig. 1A). The final enzyme preparation appeared to be homogenous, as analyzed via SDS/PAGE (Fig. 1B). The molecular mass of the enzyme was estimated to be 45 kDa via gel filtration (data not shown) and 47.2 kDa via SDS/PAGE (Fig. 1B). These results indicate that enzyme was a single subunit protein.

**Table 1:** Purification of LOD from the NUK‐21 strain

Step	Volume (mL)	Total protein (mg)	Total activity (unit)^a^	Specific activity (U·mg^−1^)^a^	Yield (%)	Purification (fold)
Crude extract	980	418	288.2	0.7	100	1.0
(NH_4_)_2_SO_4_ precipitation	1160	274	281.5	1.0	97.7	1.4
Phenyl‐650M	378	113	266.8	2.4	92.6	3.4
HW‐50	118	7.5	21.7	2.9	7.5	4.1
Ultrogel‐HA	192	2.9	15.5	5.3	5.4	7.6

a Activity was determined using the peroxidase‐4AA method.

### Enzyme as glycoprotein

The enzyme showed positive results when stained with periodic acid–Schiff's reagent in SDS/PAGE. This result establishes that the LOD is a glycoprotein (data not shown).

### Absorption spectrum and prosthetic group

Oxidases usually have a coenzyme to assist the electron transfer. Riboflavins, hemes or metals (such as copper ions) are commonly coenzymes and can be detected with a visible‐light spectral scan. The concentrated purified enzyme appeared colorless and transparent. In addition, the UV‐visible spectrum of LOD showed no signals. The metal content was determined quantitatively via ICP‐MS; the enzyme contained 2 mol of Zn^2+^ per mole of enzyme protein, with an estimated molecular weight of 47.2 kDa. A major finding was that the sugar oxidase contained zinc ions as coenzyme. The $a_{1\mathrm{cm}\;280\mathrm{nm}}^{1\%}$ value of NUK‐21 LOD was calculated to be 7.58 compared to those of other oxidases containing colorful coenzymes, which was consistent with NUK‐21 LOD did not emitting a signal on the UV‐visible absorption spectrum.

### Optimal reaction pH and pH stability

**Figure 2:**
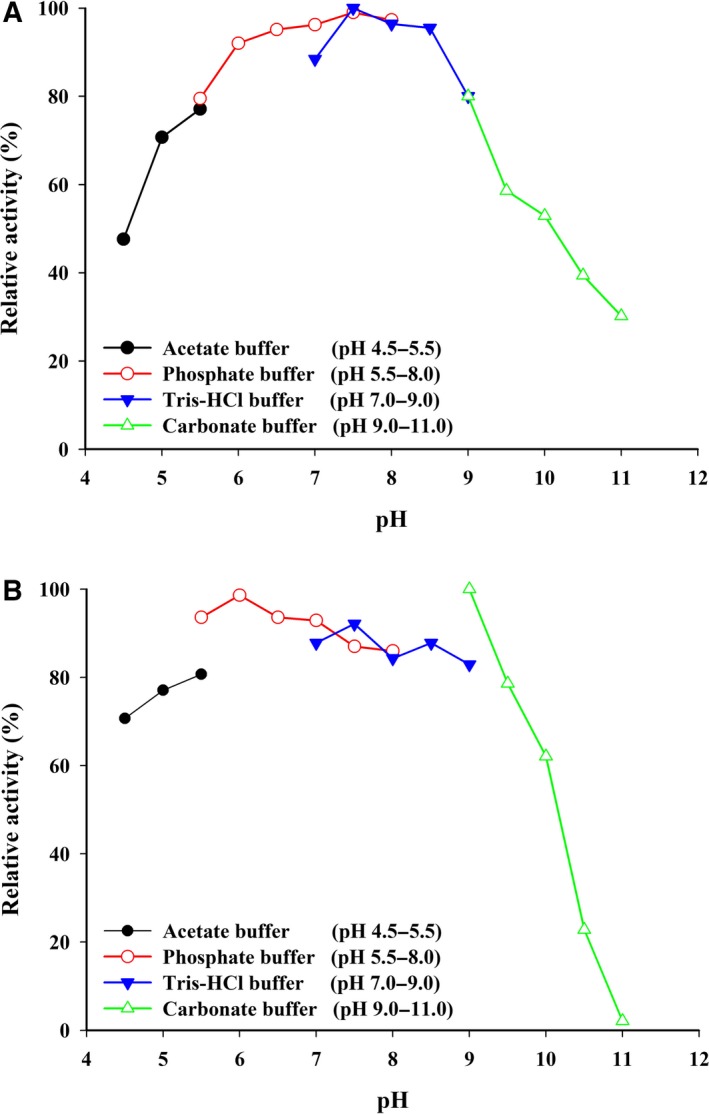
Effects of pH on LOD activity and stability. (A) Enzyme activity was assayed in various buffers at the pH values indicated by oxygraph method, as described in the text. (B) Enzyme (15 μg·mL^−1^) was incubated in various buffers at the pH values indicated. After incubation at 30 °C for 1 h, the residual activity was estimated at pH 7.8 using the peroxidase‐4AA method, as described in the text. The buffer systems (50 mm) used were acetate buffer (pH 4.5–5.5) (●), phosphate buffer (pH 5.5–8.0) (○), Tris–HCl buffer (pH 7.0–9.0) (▼) and carbonate buffer (pH 9.0–11.0) (▵).

The pH dependency of the enzyme activity was examined using the oxygraph method. When the oxygen consumption rate was measured with an oxygraph under various pH conditions, an optimal reaction range of pH 6.0–9.0 was observed, with the highest reactivity at pH 7.5. The activity dropped to less than 30% at a pH of more than 11.0 (Fig. 2A). The pH stability was investigated by incubating the enzyme at 30 °C for 1 h under various pH conditions. As shown in Fig. 2B, the enzyme activity measured using the peroxidase‐4AA method was highly stable at pH 6.0–9.0 and dropped sharply at higher pH levels.

### Optimal reaction temperature and thermal stability

**Figure 3:**
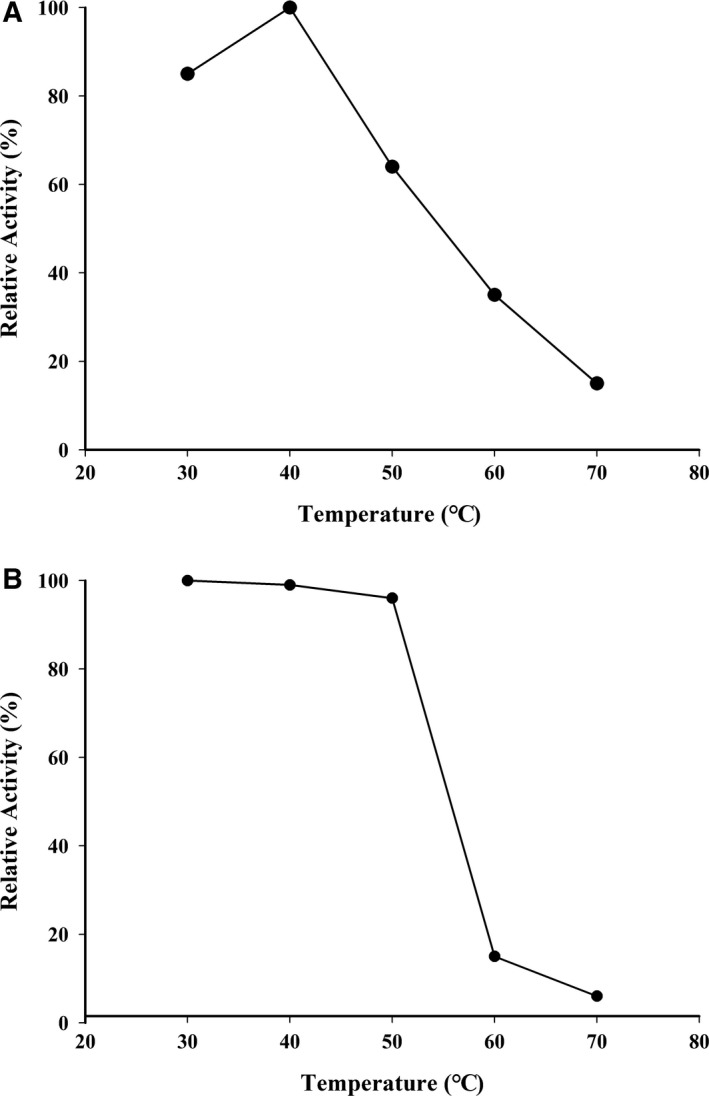
Effects of temperature on LOD activity and stability. (A) Enzyme activity was assayed by the oxygraph method, as described in the text, at various temperatures in buffer A. (B) Enzyme (15 μg·mL^−1^ in buffer A) was incubated at the temperature indicated for 1 h, and the remaining activity was estimated at 30 °C using the peroxidase‐4AA method, as described in the text.

As shown in Fig. 3A, the optimal reaction temperature for lactose oxidation under the standard oxygraph assay conditions varying from 30 °C to 50 °C was 40 °C. Thermal stability was investigated by incubating the enzyme in buffer A at a designated temperature for 1 h. The enzyme was stable up to 50 °C (Fig. 3B).

### Amino‐terminal amino acid sequence analysis

The *N*‐terminal amino acid sequence of the residues of the purified LOD was determined via gas‐phase sequencing. No signal was detected. This finding indicated that the *N*‐terminal amino acid residue of LOD was blocked by glycosylation or acetylation.

### Specificity for substrates and electron acceptors

The ability of LOD to oxidize various sugars was investigated in buffer A via the peroxidase‐4AA method (Table 2). None of the tested monosaccharides and sugar alcohols, including glucose, fructose, galactose, mannose, sorbitol, mannitol and xylitol, were oxidized. Lactose (gal‐β1,4‐glc) and cellobiose (glc‐β1,4‐glc), the disaccharides in which the two sugar units are joined by β1,4‐glycosidic links, were the preferred substrates. Maltose (glc‐α1,4‐glc), the disaccharide with α‐1,4‐glycosidic link, was a relatively poor substrate. If the reactivity of LOD toward lactose was considered as 100%, its relative activity toward cellobiose and maltose could reach 84% and 4%, respectively.

**Table 2:** Substrate specificity of LOD

Substrate	Relative activity (%)	*K*_m_ (mm)	*k*_cat_ (s^−1^)	*k*_cat_/*K*_m_ (s^−1^·mm ^−1^)
Lactose	100	0.076	97.1	1278
Cellobiose	83	0.063	71.5	1135
Maltose	4	22	65.6	3
Sucrose	0	–	–	–
Galactose	0	–	–	–
Glucose	0	–	–	–
Fructose	0	–	–	–
Xylose	0	–	–	–
Xylitol	0	–	–	–
Sorbitol	0	–	–	–

Enzyme activity was measured using the peroxidase‐4AA method. –, not reacted.

The *K*_m_ and *k*_cat_ values for the three disaccharides substrates were estimated using Hanes–Woolf plots. The apparent *K*_m_ values for lactose, cellobiose and maltose were 0.076, 0.063 and 22 mm, and the *k*_cat_ values were 97.1, 71.5 and 65.6 s^−1^, respectively. Notable substrate inhibition was observed in kinetic studies with lactose and cellobiose as substrates at a concentration higher than 1 mm. The *k*_cat_/*K*_m_ value for maltose (α1,4‐linking) was approximately 400 times smaller than that for lactose (β1,4‐linking).

In general, this novel LOD oxidized disaccharides with a glucose unit on the reducing end and each sugar unit joined by α or β‐1,4 linkage. The specificity for electron acceptors other than that for O_2_ was examined using lactose as an electron donor substrate. The enzyme did not reduce other acceptors, such as DCIP, cytochrome *c*, ferric cyanide, methylene blue or FeCl_3_, which had a favorable effect on other sugar‐oxidizing enzymes.

### Effect of metals

The purified enzyme was added to buffer A containing different 1 mm metal solutions and incubated in a water bath at 30 °C for 1 h. The effects of various metal ions and metal‐chelating agents on the enzyme activity were investigated. As shown in Table 3, most of the metal ions had little effect on the LOD activity, although both Fe^2+^ and Sn^2+^ inhibited the enzyme activities and reduced them to 61%. The LOD activity was increased by Hg^2+^ to 154%. For most enzymes, Hg^2+^ acts as an inhibitor. However, Hg^2+^ increased the activity of the novel LOD by approximately 50%.

**Table 3:** Effect of metal ions on LOD activity

Metal ions (1 mm)	Relative activity (%)
Control	100
AgNO_3_	108
CaCl_2_	110
CdSO_4_	100
CoCl_2_	110
CuSO_4_	97
FeSO_4_	61
FeCl_3_	103
HgCl_2_	154
NiSO_4_	100
PbCl_2_	90
SnCl_2_	61

Enzyme (15 μg) was incubated in buffer A containing various metal ions for 1 h at 30 °C. The residual activities were assayed by the oxygraph method.

### Effect of chemicals

As shown in Table 4, the enzyme reaction was activated by *N*‐ethylmaleimide, EDTA and NaN_3_. Only 2‐bromo‐4′‐nitroacetophenol had a significant inhibitory effect on LOD. The most potent inhibitor among the histidine group‐modifying reagents, 2‐bromo‐4′‐nitroacetophenol, inhibited the enzyme activity competitively with respect to the substrate lactose. The inhibition constant (*K*_i_) for 2‐bromo‐4′‐nitroacetophenol was found to be 0.086 mm, which is similar to the *K*_m_ value of LOD for lactose (0.076 mm). The 2‐bromo‐4′‐nitroacetophenol demonstrated non‐competitive inhibition of the enzyme reaction by blocking the histidine involved in the enzymes.

**Table 4:** Effect of chemicals on LOD activity

Chemicals	Concentration (mm)	Relative activity (%)
Control group	1	100
2,4′‐dibromoacetophenone	1	99
2‐bromo‐4′‐nitroacetophenol	1	88
NaN_3_	1	131
EDTA	1	136
*N*‐ethylmaleimide	1	140
1,10‐phenanthroline	1	99
H_2_O_2_	1	100
PMSF	1	105
2‐bromo‐4′‐nitroacetophenol	4	53

Enzyme (15 μg) was incubated in buffer A containing various chemicals for 1 h at 30 °C. The residual activities were assayed by the oxygraph method.

### Reaction product

**Figure 4:**
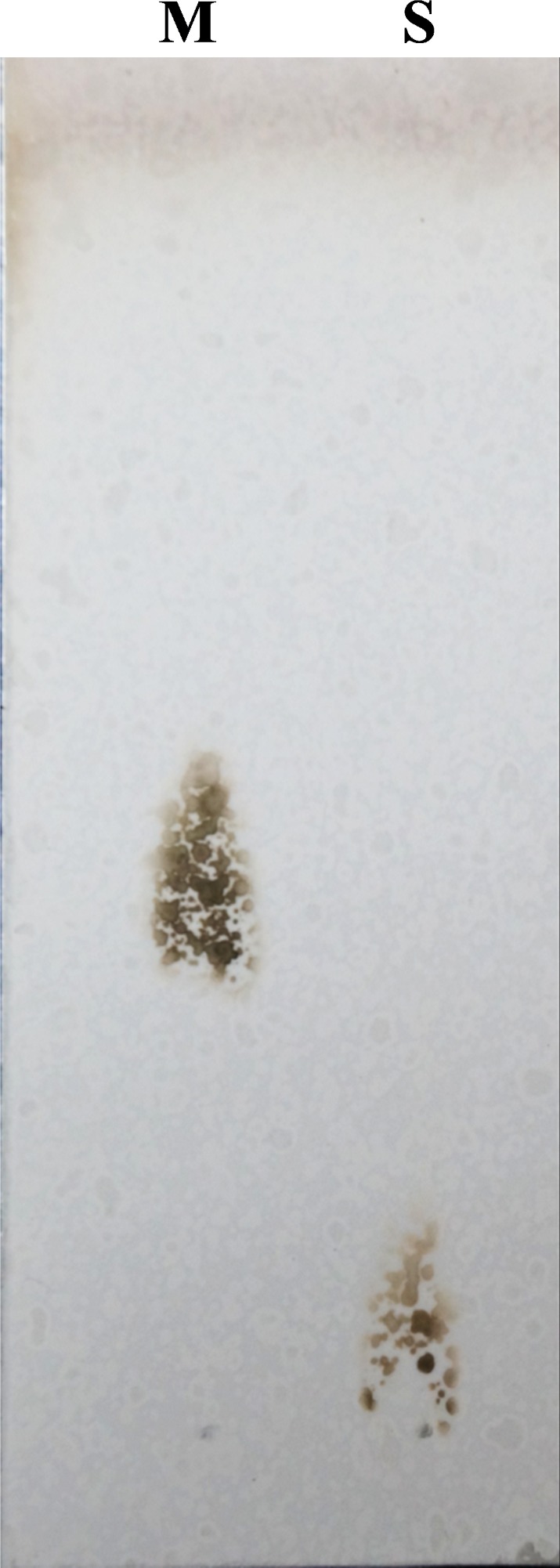
TLC analysis of the product. Lane M, 50 μg lactose; lane S, 50 μg of complete conversion product from lactose.

The reaction product of LOD was studied with lactose as the substrate. After the aerobic incubation of lactose with an excess amount of LOD in the presence of a catalase at 30 °C for 6 h, the reaction products were examined via TLC on silica gel 60 plates (Fig. 4). Only one new compound was observed with the same *R*_f_ value as that of LBA. This compound was eluted from the thin‐layer plate, purified using HPLC using a μ‐Spherogel column (8.0 mm × 300 mm) (Beckman Instruments, Palo Alto, CA, USA) at 60 °C with deionized water as a eluent (1 mL·min^−1^), and then analyzed by negative ion electrospray ionization–mass spectrometry. It exhibited a [M–H]^−^ ion at *m*/*z* 357.11, indicating a molecular weight of 358 Da, equal to that of the LBA standard. The ^13^C nuclear magnetic resonance spectrum analysis indicated that the position of the gluconic acid moiety of LBA shifted to 179.23 ppm, confirming that the enzyme reaction product was LBA.

## Discussion

Recently, we adopted a selective and rapid *in situ* assay for detecting H_2_O_2_‐producing LOD produced by soil fungi. The LOD‐producing strain NUK‐21 was identified as *M. flexuosum* based on the morphology of the mycelium, spores and rDNA sequences. *Myrmecridium flexuosum* was a new published species and a few studies have reported on oxidase production, especially sugar oxidase production. In addition, many studies on sugar oxidoreductase produced from mycelium fungi have rarely reported it to contain Zn^2+^.

LOD is a glycoprotein with a high sugar content. Despite application of 55% saturation with ammonium sulfate, the enzyme could not be extracted without precipitation. LOD purification from the initial crude is difficult because it tends to combine with other cellular components to form macromolecules. This enzyme was eluted near the void volume in a Fractogel HW‐55 column (exclusion limit 7 × 10^5^ Da). This suggested that LOD has a strong tendency to aggregate. The same phenomenon was observed in the PAGE and chromatography analysis results. In the native PAGE experiment, a major portion of the purified LOD failed to penetrate into the separating gel. Similarly, the enzyme aggregated when subjected to gel filtration chromatography with the absence of 0.2% SDS. A similar result was reported for other glycoproteins, which can then be purified via gel filtration in the presence of a depolymerizing agent such as SDS 26.

The lower yield and higher resolution of purification were attributed to Fractogel HW‐50 column chromatography with 0.2% SDS in the purification steps. Because the aggregations of LOD and other proteins were very high, separation of the enzyme by chromatography was difficult. However, efficient separation was achieved by adding SDS during Fractogel HW‐50 (exclusion limit 8 × 10^4^ Da) column chromatography. The 0.2% SDS added during Fractogel HW‐50 column chromatography was able to separate active fractions of the LOD into two parts: the major aggregated fractions and minor non‐aggregate fractions. These fractions were segregated based on the molecular sizes of the proteins. The major aggregations of proteins could be observed during SDS/PAGE with other proteins and LOD (Fig. 1A).

Based on the cofactor species, substrate specificity of the enzyme found in the present study has not been reported in the literature. As shown in Table 5, the LOD purified from the wheat bran culture of *M. flexuosum* NUK‐21 is a new type of sugar oxidase. This enzyme does not contain a flavin prosthetic group, unlike glucose oxidase, which contains FAD as the prosthetic group 27 and galactose oxidase with Cu as the cofactor 28. The most unexpected characteristic of this enzyme is that its cofactor is notably different from other fungi sugar oxidases, including those listed in Table 5. The ICP‐MS analysis result supports the conclusion that the molecular LOD is a zinc enzyme containing two Zn^2+^ ions based on the molar ratio of Zn/LOD. A potent histidine group–modifying reagent 2‐bromo‐4′‐nitroacetophenol inhibited the enzyme activity competitively with respect to the substrate lactose. The Zn^2+^ ion can bind to histidine noncovalently. Thus, 2‐bromo‐4′‐nitroacetophenol inhibits the enzyme reaction by blocking the histidine involved in the enzymes. Further research aiming to identify the Zn‐binding motif on the LOD via protein sequencing is underway.

**Table 5:** Comparison of the properties of various sugar oxidoreductases

Enzyme	Strain	Molecular mass (kDa)	Prosthetic group	Substrate specificity (%)		
				Lactose	Cellobiose	Maltose
LOD	*Myrmecridium flexuosum*	47	Zn	100	83	4
COX	*Microdochium nivale*	55	FAD	52	100	ND
PCOX	*Paraconniothyrium* sp	54	FAD	–	–	–
COOX	*Sarocladium oryzae*	51	FAD	83	100	13
GOOX	*Acremonium strictum*	61	FAD	64	47	100
CBG	*Phanerochaete chrysosporium*	58	FAD	–	–	–

Zn^2+^ was discovered as a cofactor in several enzymes, including carbonic anhydrase 29, phospholipase C 30, aminopeptidase 31 and alcohol dehydrogenase 32, via catalytic hydrolysis or closely related transfer reactions. Enzyme‐bound Zn^2+^ can function as a catalyst by forming complexes with the substrate, either by expanding its coordination domain or by exchanging a ligand. Formation of enzyme‐Zn^2+^‐substrate bonds can position the substrate or polarize its electron distribution to facilitate further reactions. Most zinc‐containing enzymes are hydrolases, in which water or hydroxide coordinated to Zn^2+^ is the nucleophile in many hydrolysis reactions 33. The catalytic mechanisms of Zn^2+^ in enzymes are not involved in the oxidation–reduction reactions of Zn^2+^ ion even in the alcohol dehydrogenase 32. Zinc ion was considered to stabilize the quaternary structure of the enzyme via the formation of bridges between the monomeric to an enzymatically active tetramer 34. It did not directly participate in electron transfer. The major oxidation–reduction reactions were carried out by nicotinamide adenine dinucleotide (NAD^+^), and not Zn^2+^ ion. Currently, only rarely reported sugar oxidases contain Zn^2+^ as a cofactor in the oxidation of sugars to aldonic acids. Interestingly, our discovered *M. flexuosum* NUK‐21 enzyme contains only a Zn^2+^ prosthetic group and no other cofactors. Only glucose dehydrogenase contains Zn^2+^ as a cofactor 35, although it is not an oxidase. The role of Zn^2+^ in the catalytic process is not clear and additional studies are required.

Metal ions generally form complexes with ionized carbohydrates and change both their solubility and behavior at the interface. However, the inhibition of the enzyme might involve binding of the catalytic site or alteration of the interface properties. Most enzyme activities shown in Table 3 were not inhibited by the metal ions. This may be because the relationship among carbohydrates on the enzyme was highly ionized. Notably, the enzyme activity was not inhibited by Fe^3+^ but was significantly inhibited by Fe^2+^. The 1 mm of Fe^2+^ in the 50 mm of Tris buffer (pH 7.8) is colorless, and the Fe^3+^ is yellow. After 1 mm of Fe^2+^ ions was incubated with the enzymes, the mixture solution turned yellow (data not shown). Zn^2+^ cannot be reduced into Zn by the Fe^2+^. This might be because Fe^2+^ reacted with enzyme and the enzyme activity was affected. However, the phenomenon regarding LOD activity enhancement by Hg^2+^ has rarely been reported and remains unexplained.

Table 5 presents a summary of the general properties of LOD obtained from *M. flexuosum* NUK‐21 and those of COX obtained from *M. nivale* 20, 36, PCOX obtained from *Paraconniothyrium* sp. 21, cellooligosaccharide oxidase (COOX) obtained from *S. oryzae* 19, GOOX obtained from *Acremonium strictum* T1 37 and cellobiose quinone dehydrogenase (CBG) obtained from *P. chrysosporium* 38. All of these five enzymes are monomeric proteins with a molecular weight in the range 51–61 kDa and the molecular weight of the enzyme in the present study is 48 kDa. LOD contains Zn as the prosthetic group, whereas the other five enzymes contain only FAD.

The other notable difference between the novel LOD and other oxidases concerns substrate specificity. The specificity of other previous carbohydrate oxidases to disaccharides, especially to cellobiose, was higher than that to lactose, although the specificity of LOD in the present study to lactose was higher than that to cellobiose (100 : 83). The substrate specificity of the novel LOD is the highest compared to the other oxidases in the literature. Our enzyme had the highest activity toward lactose, followed by cellobiose and maltose. No sugar oxidases have been reported to exhibit reactivity toward disaccharides. Thus, the present study provides the first report with respect to the discovery of a true LOD.

In summary, our newly discovered the LOD differs from previously reported ones in the several aspects: molecular weight, substrate specificity and prosthetic group. The results strongly suggest that *M. flexuosum* NUK‐21 produces a novel LOD. LOD is considered more suitable for the conversion lactose to produce LBA as a result of its low *K*_m_ and high *k*_cat_ values. Because of the high reactivity and specificity of LOD toward these sugars, this enzyme can also be used for lactose and cellobiose quantification. Given its resistance to H_2_O_2_, it should prove useful in LBA production. LOD is expected to be applied in pharmaceutical and food industries in the future.

## Conflicts of interest

The authors declare no conflict of interest.

## Author contributions

S‐FL made substantial contributions to the study conception, design and organization of the study, participated in interpreting the data, drafted the article, revised it critically for important intellectual content, and gave final approval to the version submitted for publication. C‐KL and Y‐PC participated in all of the experiments and made contributions regarding both the acquisition and analysis of data.

## References

[1] Gutiérrez LF , Hamoudi S and Belkacemi K (2012) Lactobionic acid: a high value‐added lactose derivative for food and pharmaceutical applications. Int Dairy J 26, 103–111.

[2] Yang BY and Montgomery R (2005) Oxidation of lactose with bromine. Carbohydr Res 340, 2698–2705. 16202397 10.1016/j.carres.2005.05.025

[3] Alonso S , Rendueles M and Díaz M (2012) Role of dissolved oxygen availability on lactobionic acid production from whey by *Pseudomonas taetrolens* . Bioresour Technol 109, 140–147. 22310213 10.1016/j.biortech.2012.01.045

[4] Alonso S , Rendueles M and Díaz M (2013) Feeding strategies for enhanced lactobionic acid production from whey by *Pseudomonas taetrolens* . Bioresour Technol 134, 134–142. 23500570 10.1016/j.biortech.2013.01.145

[5] Miyamoto Y , Ooi T and Kinoshita S (2000) Production of lactobionic acid from whey by *Pseudomonas* sp. LS13–1. Biotechnol Lett 22, 427–430.

[6] Malvessi E , Carra S , Pasquali FC , Kern DB , da Silveira MM and Ayub MAZ (2013) Production of organic acids by periplasmic enzymes present in free and immobilized cells of *Zymomonas mobilis* . J Ind Microbiol Biotechnol 40, 1–10. 23053345 10.1007/s10295-012-1198-6

[7] Pedruzzi I , da Silva EAB and Rodrigues AE (2011) Production of lactobionic acid from lactose/fructose substrate using GFOR/GL enzymes from *Zymomonas mobilis* cells: a kinetic study. Enzyme Microb Technol 49, 183–191. 22112407 10.1016/j.enzmictec.2011.04.017

[8] Murakami H , Seko A , Azumi M , Ueshima N , Yoshizumi H and Nakano H (2003) Fermentative production of lactobionic acid by *Burkholderia cepacia* . J Appl Glycosci 50, 117–120.

[9] Murakami H , Seko A , Azumi M , Kiso T , Kiryu T and Kitahata S (2006) Microbial conversion of lactose to lactobionic acid by resting cells of *Burkholderia cepacia* No. 24. J Appl Glycosci 53, 7–11.

[10] Kiryu T , Yamauchi K , Masuyama A , Ooe K , Kimura T and Kiso T (2012) Optimization of lactobionic acid production by *Acetobacter orientalis* isolated from Caucasian fermented milk, “Caspian Sea yogurt”. Biosci Biotechnol Biochem 76, 361–363. 22313756 10.1271/bbb.110608

[11] Hilt W , Pfleiderer G and Fortnagel P (1991) Glucose dehydrogenase from *Bacillus subtilis* expressed in *Escherichia coli*. I: purification, characterization and comparison with glucose dehydrogenase from *Bacillus megaterium* . Biochim Biophys Acta 1076, 298–304. 1900201 10.1016/0167-4838(91)90281-4

[12] Leitner C , Volc J and Haltrich D (2001) Purification and characterization of pyranose oxidase from the white rot fungus *Trametes multicolor* . Appl Environ Microbiol 67, 3636–3644. 11472941 10.1128/AEM.67.8.3636-3644.2001 PMC93065

[13] Shin KS , Youn HD , Han YH , Kang SO and Hah YC (1993) Purification and characterisation of d‐glucose oxidase from white‐rot fungus *Pleurotus ostreatus* . Eur J Biochem 215, 747–752. 8354282 10.1111/j.1432-1033.1993.tb18088.x

[14] Groen BW , Vries SD and Duine JA (1997) Characterization of hexose oxidase from the red seaweed *Chondrus crispus* . Eur J Biochem 244, 858–861. 9108257 10.1111/j.1432-1033.1997.00858.x

[15] Hansen OC and Stougaard P (1997) Hexose oxidase from the red alga *Chondrus crispus* . J Biol Chem 272, 11581–11587. 9111074 10.1074/jbc.272.17.11581

[16] Avigad G , Amaral D , Asensio C and Horecker BL (1962) The d‐galactose oxidase of *Polyporus circinatus* . J Biol Chem 237, 2736–2743. 13863403

[17] Henriksson G , Johansson G and Pettersson G (2000) A critical view of cellobiose dehydrogenase. J Biotechnol 78, 93–113. 10725534 10.1016/s0168-1656(00)00206-6

[18] Morpeth FF and Jones GD (1986) Resolution, purification and some properties of the multiple forms of cellobiose quinone dehydrogenase from the white‐rot fungus *Sporotrichum pulverulentum* . Biochem J 236, 221–226. 3790072 10.1042/bj2360221 PMC1146809

[19] Lee MH , Lai WL , Lin SF , Liu Y , Hsu YH and Tsai YC (2006) Purification and characterization of a novel cellooligosaccharide oxidase from rice pathogen *Sarocladium oryzae* . Enzyme Microb Technol 39, 85–91.

[20] Xu F , Golightly EJ , Fuglsang CC , Schneider P , Duke KR , Lam L , Christensen S , Brown KM , Jùrgensen CT and Brown SH (2001) A novel carbohydrate: acceptor oxidoreductase from *Microdochium nivale* . Eur J Biochem 268, 1136–1142. 11179980 10.1046/j.1432-1327.2001.01982.x

[21] Kiryu T , Nakano H , Kiso T and Murakami H (2008) Purification and characterization of a carbohydrate: acceptor oxidoreductase from *Paraconiothyrium* sp. that produces lactobionic acid efficiently. Biosci Biotechnol Biochem 72, 833–841. 18323642 10.1271/bbb.70701

[22] Danneel HD , Ullrich M and Giffhorn F (1992) Goal‐oriented screening method for carbohydrate oxidases produced by filamentous fungi. Enzyme Microb Technol 14, 898–903.

[23] Laemmli UK (1970) Cleavage of structural protein during the assembly of the head of bacteriophage T4. Nature 227, 680–685. 5432063 10.1038/227680a0

[24] Zaccharius RM , Zell TE , Morrison JH and Woodlock JJ (1969) Glycoprotein staining following electrophoresis on acrylamide gels. Anal Biochem 31, 148–152. 10.1016/0003-2697(69)90383-2 4183001

[25] Lowry OH , Rosebrough NJ , Farr AL and Randall RJ (1951) Protein measurement with the Folin phenol reagent. J Biol Chem 193, 265–275. 14907713

[26] Campbell KP and MacLennan D (1981) Purification and characterization of the 53,000‐dalton glycoprotein from the sarcoplasmic reticulum. J Biol Chem 256, 4626–4632. 6260806

[27] Keilin D and Hartree EF (1946) Prosthetic group of glucose oxidase (Notatin). Nature 157, 801. 10.1038/157801a0 20987676

[28] Amaral D , Bernstein L , Morse D and Horecker BL (1963) Galactose oxidase of *Polyporus cireinatus*: a copper enzyme. J Biol Chem 238, 2281–2284. 14012475

[29] Lindskog S and Coleman JE (1973) The catalytic mechanism of carbonic anhydrase. Proc Natl Acad Sci U S A 70, 2505–2508. 4200327 10.1073/pnas.70.9.2505 PMC427044

[30] da Graça Thrige D , Buur JR and Jørgensen FS (1997) Substrate binding and catalytic mechanism in phospholipase C from *Bacillus cereus:* a molecular mechanics and molecular dynamics study. Biopolymers 42, 319–336. 9279125 10.1002/(SICI)1097-0282(199709)42:3<319::AID-BIP5>3.0.CO;2-P

[31] Chevier B , Schalk C , D'Orchymont H , Rondeau J‐M , Moras D and Tarnus C (1994) Crystal structure of *Aeromonas proteolytica* aminopeptidase: a prototypical member of the co‐catalytic zinc enzyme family. Structure 2, 283–291. 8087555 10.1016/s0969-2126(00)00030-7

[32] Vallee BL and Hoch FL (1955) Zinc, a component of yeast alcohol dehydrogenase. Proc Natl Acad Sci U S A 41, 2505–2508. 10.1073/pnas.41.6.327 PMC528091 16589675

[33] Coleman JE (1998) Zinc enzyme. Curr Opin Chem Biol 2, 222–234. 9667939 10.1016/s1367-5931(98)80064-1

[34] Kagi JHR and Vallee BL (1960) The role of zinc in alcohol dehydrogenase: V. The effect of metal‐binding agents on the structure of the yeast alcohol dehydrogenase molecule. J Biol Chem 235, 3188–3192. 13750715

[35] John J , Crennel SJ , Hough DW , Danson MJ and Taylor GL (1994) The crystal structure of glucose dehydrogenase from *Thermoplasma acidophilum* . Structure 2, 385–393. 8081754 10.1016/s0969-2126(00)00040-x

[36] Nordkvist M , Nielsen PM and Villadsen J (2007) Oxidation of lactose to lactobionic acid by a *Microdochium nivale* carbohydrate oxidase: kinetic and operational stability. Biotechnol Bioeng 97, 694–707. 17154316 10.1002/bit.21273

[37] Lin SF , Yang TY , Inukai T , Yamasaki M and Tsai YC (1991) Purification and characterization of a novel glucooligosaccharide oxidase from *Acremonium strictum* T1. Biochim Biophys Acta 1118, 41–47. 1764476 10.1016/0167-4838(91)90439-7

[38] Morpeth FF (1985) Some properties of cellobiose oxidase from the white‐rot fungus *Sporotrichum pulverulentum* . Biochem J 228, 557–564. 2992449 10.1042/bj2280557 PMC1145023

